# Diagnostic utility of clinicodemographic, biochemical and metabolite variables to identify viable pregnancies in a symptomatic cohort during early gestation

**DOI:** 10.1038/s41598-024-61690-3

**Published:** 2024-05-15

**Authors:** Christopher J. Hill, Marie M. Phelan, Philip J. Dutton, Paula Busuulwa, Alison Maclean, Andrew S. Davison, Josephine A. Drury, Nicola Tempest, Andrew W. Horne, Eva Caamaño Gutiérrez, Dharani K. Hapangama

**Affiliations:** 1 Department of Women’s and Children’s Health, Centre for Women’s Health Research, Institute of Life Course and Medical Sciences, University of Liverpool, Member of Liverpool Health Partners, Liverpool, L8 7SS UK; 2 High Field NMR Facility, Liverpool Shared Research Facilities, University of Liverpool, Liverpool, L69 7TX UK; 3 Department of Biochemistry and Systems Biology, Institute of Systems, Molecular and Integrative Biology, University of Liverpool, Liverpool, L69 7ZB UK; 4 Liverpool Women’s Hospital NHS Foundation Trust, Member of Liverpool Health Partners, Liverpool, L8 7SS UK; 5 Department of Clinical Biochemistry and Metabolic Medicine, Liverpool Clinical Laboratories, Liverpool University Hospitals NHS Foundation Trust, Liverpool, L7 8SP UK; 6 Centre for Reproductive Health, Institute for Regeneration and Repair, University of Edinburgh, Edinburgh, EH16 4UU UK; 7 Computational Biology Facility, Liverpool Shared Research Facilities, University of Liverpool, Liverpool, L69 7ZB UK

**Keywords:** Ectopic pregnancy, Miscarriage, Early pregnancy, Biomarkers, Metabolomics, Machine learning, Biomarkers, Biomarkers, Medical research, Machine learning

## Abstract

A significant number of pregnancies are lost in the first trimester and 1–2% are ectopic pregnancies (EPs). Early pregnancy loss in general can cause significant morbidity with bleeding or infection, while EPs are the leading cause of maternal mortality in the first trimester. Symptoms of pregnancy loss and EP are very similar (including pain and bleeding); however, these symptoms are also common in live normally sited pregnancies (LNSP). To date, no biomarkers have been identified to differentiate LNSP from pregnancies that will not progress beyond early gestation (non-viable or EPs), defined together as combined adverse outcomes (CAO). In this study, we present a novel machine learning pipeline to create prediction models that identify a composite biomarker to differentiate LNSP from CAO in symptomatic women. This prospective cohort study included 370 participants. A single blood sample was prospectively collected from participants on first emergency presentation prior to final clinical diagnosis of pregnancy outcome: LNSP, miscarriage, pregnancy of unknown location (PUL) or tubal EP (tEP). Miscarriage, PUL and tEP were grouped together into a CAO group. Human chorionic gonadotrophin β (β-hCG) and progesterone concentrations were measured in plasma. Serum samples were subjected to untargeted metabolomic profiling. The cohort was randomly split into train and validation data sets, with the train data set subjected to variable selection. Nine metabolite signals were identified as key discriminators of LNSP versus CAO. Random forest models were constructed using stable metabolite signals alone, or in combination with plasma hormone concentrations and demographic data. When comparing LNSP with CAO, a model with stable metabolite signals only demonstrated a modest predictive accuracy (0.68), which was comparable to a model of β-hCG and progesterone (0.71). The best model for LNSP prediction comprised stable metabolite signals and hormone concentrations (accuracy = 0.79). In conclusion, serum metabolite levels and biochemical markers from a single blood sample possess modest predictive utility in differentiating LNSP from CAO pregnancies upon first presentation, which is improved by variable selection and combination using machine learning. A diagnostic test to confirm LNSP and thus exclude pregnancies affecting maternal morbidity and potentially life-threatening outcomes would be invaluable in emergency situations.

## Introduction

Pelvic pain and vaginal bleeding are common in early pregnancy. Up to 30% of women will experience these symptoms during the first trimester^1,2^ and require a medical assessment and elimination of differential diagnosis that includes miscarriage, ectopic pregnancy (EP) or pregnancy of unknown location (PUL). Approximately 15% of known correctly sited pregnancies end in miscarriage and can cause significant morbidity if heavy bleeding or infection occurs^3^. A further 1–2% of pregnancies are located outside the endometrial cavity, with the majority (~ 98%) implanting in the fallopian tubes; these EPs are the leading cause of maternal mortality in the first trimester^4,5^. PUL describes a pregnancy that cannot be located on transvaginal ultrasonography (TVS); 5–42% of early pregnancy scans fall into this category^6^. Those pregnancies that are assigned as PULs at first emergency presentation have clinically heterogenous outcomes; their final diagnosis includes (i) a live normally-sited pregnancy (LNSP), (ii) a non-viable normally-sited pregnancy (NVNSP), (iii) an EP, (iv) a failed PUL or (v) a persistent PUL^7^. Considering the possible consequences of each pregnancy outcome, early and accurate identification upon first presentation of symptomatic women with a LNSP from any other pregnancy outcome will reduce the diagnostic burden, as well as maternal morbidity and mortality.

Diagnosis of an adverse early pregnancy outcome in clinical practice depends on a combination of ultrasonographic, biochemical, clinical, and sometimes surgical assessment. TVS at first presentation is inconclusive in approximately 4–40% of cases, likely due to (i) a LNSP before the detection limit of TVS, (ii) failure before gestation sac formation or (iii) being an EP^8^. The detection limit of a LNSP by TVS is ~ 6 weeks of gestation^9^, however, a significant proportion of pregnancies are unplanned^10^. Therefore, dating of the pregnancy from last menstrual period is frequently unreliable, which further complicates diagnosis at first emergency presentation. Human chorionic gonadotrophin β (β-hCG) and progesterone are used in routine clinical practice to monitor pregnancies not located on TVS; doubling of β-hCG over 48 h is indicative of a LNSP^11^, and TVS should be able to locate a LNSP with a serum β-hCG level ≥ 1500 IU/L^12^. An initial progesterone level cutoff of ≤ 2 nmol/L has been shown to accurately categorise low risk PULs^13^. However, serial β-hCG concentrations are required to accurately differentiate LNSPs from adverse outcomes^8^. Such management runs the risk of tubal rupture in women with EP, and unwarranted anxiety and clinical intervention in others with LNSP^14^. Since most symptomatic patients will have a LNSP, their rapid identification at first presentation and reassurance will allow focussing of the available diagnostic resources on women with a high risk of conditions that can be associated with maternal morbidity and mortality. A plethora of novel blood and urine biomarkers have been identified in the pursuit of improved diagnostics for miscarriage, EP and PUL^7,8,15–18^. However, none have yet been translated to routine clinical care. Few studies have assessed biomarkers for the definitive diagnosis of a LNSP in symptomatic cohorts containing multiple adverse outcomes. Therefore, there is an urgent need for a rapid, non-invasive, and single measurement test to accurately identify a LNSP, and exclude NVNSPs and EPs.

Metabolomics is the comprehensive analysis of small molecules (< 1500 Da) in biological samples such as cells, tissues and biofluids. In humans, metabolomic profiling can be used to identify disease biomarkers and elucidate metabolic pathways involved in pathological processes^19^. Nuclear magnetic resonance (NMR) spectroscopy and mass spectrometry (MS) are the two most employed techniques in metabolomic research^20,21^. NMR metabolomic analysis provides highly reproducible, global quantitation of measurable analytes in biological samples in an unbiased and non-destructive fashion^22^. Routinely collected biofluids in early pregnancy assessment, such as blood and urine, are well-established sample types for NMR metabolomic profiling and are ideal for biomarker screening with simple and minimally invasive sample collection.

The analysis of omics data and its exploitation for in-silico biomarker discovery using machine learning is not trivial, with an ongoing crisis of reproducibility in the field^23^. We have applied a state-of-the-art pipeline to detect consensus, robust signals across patients. In this study, we propose a composite biomarker of early LNSP when compared with non-viable correctly sited pregnancies and EPs (these were grouped together as combined adverse outcomes, henceforth CAO). We apply a novel machine learning pipeline for robust biomarker identification and create models that integrate untargeted ^1^H NMR small molecule profiling of serum combined with biochemical markers (progesterone, β-hCG) and patient demographics.

## Results

### Study cohort

**Figure 1:**
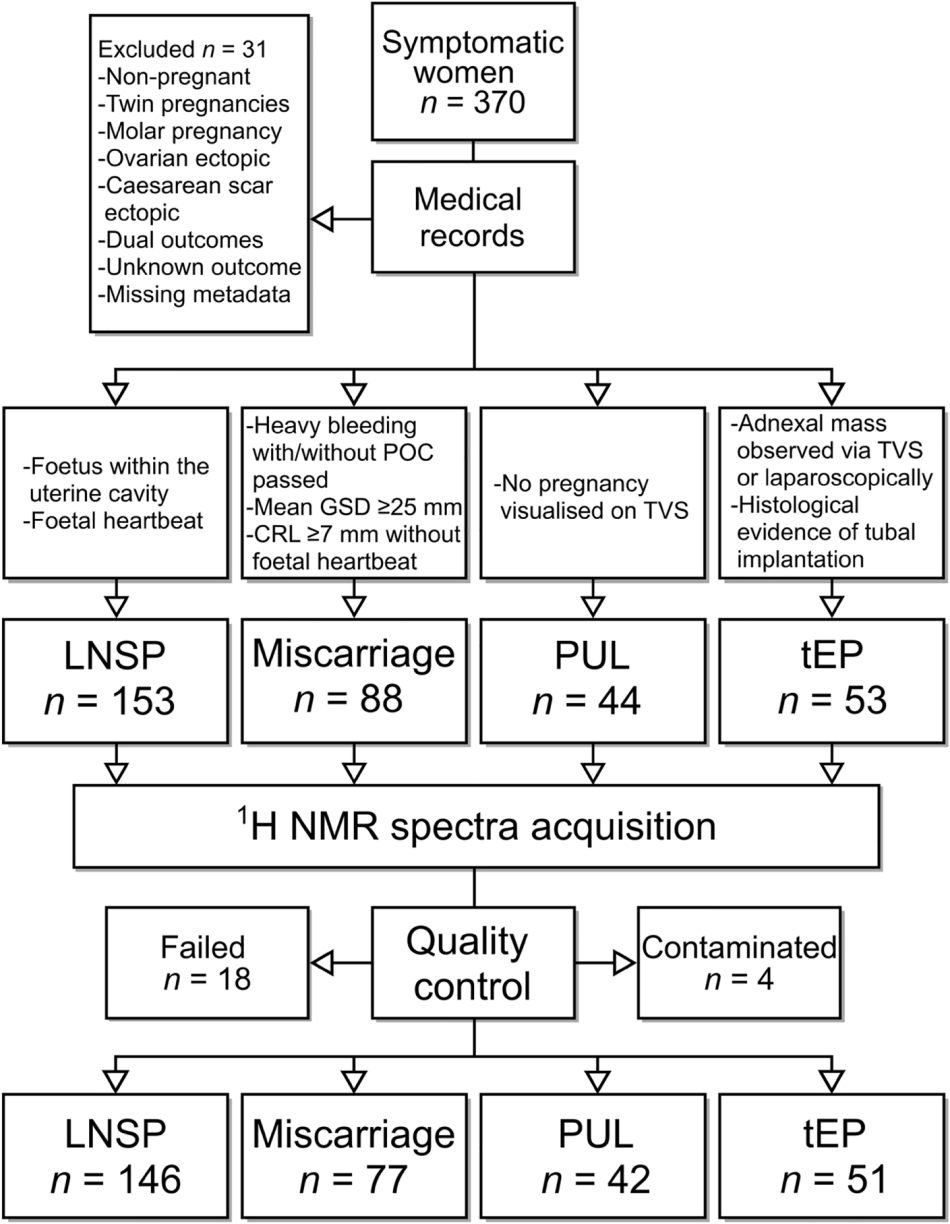
Project workflow and clinical features of pregnancy outcomes. Abbreviations: *POC* products of conception, *GSD* gestational sack diameter, *CRL* crown-rump length, *TVS* transvaginal ultrasound, *LNSP* live normally-sited pregnancy, *PUL* pregnancy of unknown location, *tEP* tubal ectopic pregnancy.

A total of 370 participants were recruited to this study. Of these participants, 31 were excluded before sample analysis (Supplementary Table 1); two were found to be non-pregnant, and two had twin pregnancies, which were excluded on the basis that they may confound metabolomic profiles of singleton pregnancies. Two participants had vanishing twin syndrome (dual outcome). It was decided to include only confirmed tEP within the ectopic group, therefore, a left ovarian EP and a caesarean section EP were excluded. Nine participants were excluded based on an unknown pregnancy outcome; these patients typically had a PUL or an early LNSP (a pregnancy within the uterine cavity which has a potential to develop normally)^24^; however, they had a termination of pregnancy before 12 weeks’ gestation or were lost to follow up. Finally, 14 participants were excluded due to missing clinicodemographic data including BMI, gestational age, and hormone levels (Supplementary Table 1). The remaining 339 participants were categorised into four pregnancy outcomes: LNSP, miscarriage, PUL and tEP (Fig. 1). Following serum spectra acquisition and quality control, 18 spectra that did not meet the minimum reporting standards were removed from further analysis. Furthermore, three spectra were found to contain EDTA, suggesting that plasma had been erroneously collected before serum during serial blood specimen acquisition and thus were excluded. Finally, one sample contained a high level of gluconic acid and was removed from further analysis (Supplementary Table 1). In total, 146 LNSPs, 77 miscarriages, 42 PULs and 51 tEPs were included in this study. Participant demographic information is summarised in Table 1. There were no significant differences in known confounding variables in metabolomics research between groups, including BMI and smoking status.

**Table 1:** Participant demographics.

	LNSP	Miscarriage	PUL	tEP	*p*-value
Number	146	77	42	51	
Age in years, median (IQR)	30 (8)	31 (10)	31 (10)	29 (10)	0.21^a^
Caucasian ethnicity, *n* (%)	127 (87.0)	63 (81.9)	36 (87.8)	41 (89.1)	0.63^b^
BMI (kg/m^2^), median (IQR)	25.8 (7.9)	25.8 (9.4)	23.6 (6.3)	24.7 (8.4)	0.13^a^
Smoker, *n* (%)	36 (24.7)	18 (23.4)	7 (17.1)	14 (27.5)	0.70^b^
Nullipara, *n* (%)	54 (37.0)	25 (32.5)	18 (43.9)	15 (29.4)	0.54^b^
Gestational age in weeks, median (IQR)	6 (2)	6 (1)	6 (2)	6 (2)	0.10^a^
β-hCG U/L, median (IQR)	9893 (42,310)	607 (3562)	100 (378)	859 (2686)	< 0.001^a^
Progesterone nmol/L, median (IQR)	57 (44)	25 (44)	12 (43)	25 (42)	< 0.001^a^

*BMI* body mass index, *β-hCG* human chorionic gonadotrophin β, *IQR* interquartile range, *LNSP* live normally-sited pregnancy, *PUL* pregnancy of unknown location and *tEP* tubal ectopic pregnancy.

^a^Kruskal–Wallis; ^b^Chi-squared.

### Plasma β-hCG and progesterone concentrations across early pregnancy outcomes

Plasma concentrations of β-hCG and progesterone were significantly higher in the LNSP group compared with the adverse outcomes of miscarriage, PUL and tEP (Table 1). Gestational age demonstrated a highly significant positive correlation (*p* < 0.001) with plasma β-hCG concentrations in the LNSP group, and weak positive correlations in the miscarriage and PUL groups. Progesterone concentration did not significantly correlate with either gestational age or β-hCG in any group (Supplementary Table 2).

### Metabolite identification

**Figure 2:**
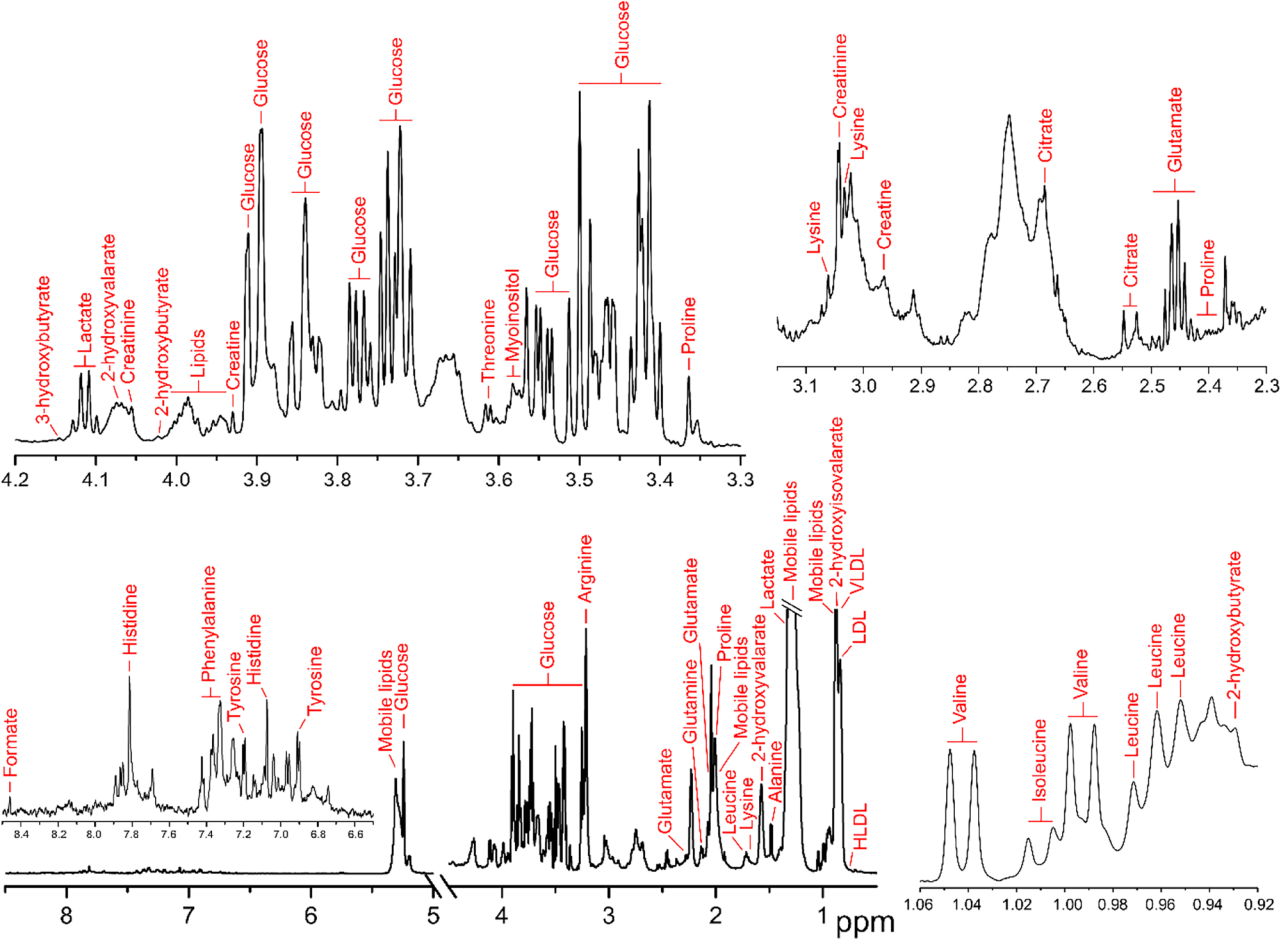
Serum metabolite annotation. Typical 700 MHz Carr–Purcell–Meiboom–Gill (CPMG) ^1^H spectra of human serum in phosphate buffer. Peaks derived from identified metabolites are annotation.

Proton spectra of serum were divided into 162 bins, of which 92 (56.8%) were assigned to a metabolite. A total of 32 unique metabolites were annotated, which included amino acids (e.g., phenylalanine, valine, leucine, isoleucine), organic acids (e.g., lactate, formate) and saccharides (e.g., glucose) (Fig. 2, Table 2). Metabolites identified using two independent, orthogonal datasets were allocated MSI level 1 assignment in accordance with best practice^25,26^ (Table 2). To remove redundant peaks arising from the same molecule, correlations were calculated between all signals originating from individual metabolites. The highest correlating peaks were then selected as most representative of a given metabolite and taken forward for statistical analysis (Table 2). The refined data set contained 102 bins in total, comprising annotated metabolites (32 bins) and unannotated signals (70 bins).

**Table 2:** Metabolites annotated in serum using Chenomx (level 2/3) or identified using in-house standards (level 1) as recommended by the Metabolomics Standard Initiative (MSI).

Database identifier	Metabolite	Reliability	Representative bin (ppm)
HMDB0000008	2-Hydroxybutyrate	MSI level 2	0.932–0.921
HMDB0000407	2-Hydroxyisovalerate	MSI level 2	0.893–0.879
HMDB0001863	2-Hydroxyvalerate	MSI level 2	1.621–1.530
HMDB0000357	3-Hydroxybutyrate	MSI level 2	1.209–1.120
HMDB0000042	Acetate	MSI level 1	1.923–1.914
HMDB0000094	Citrate	MSI level 1	2.552–2.521
HMDB0000064	Creatine	MSI level 1	3.935–3.925
HMDB0000562	Creatinine	MSI level 1	4.059–4.052
HMDB0000122	d-glucose	MSI level 1	3.741–3.732
HMDB0304356	Formate	MSI level 2	8.483–8.455
HMDB0000190	Lactate	MSI level 1	4.124–4.093
HMDB0000161	l-alanine	MSI level 1	1.500–1.464
HMDB0000517	l-arginine*	MSI level 2	1.914–1.860
			3.236–3.203
HMDB0000148	l-glutamate	MSI level 1	2.278–2.257
HMDB0000641	l-glutamine	MSI level 1	2.177–2.109
HMDB0000123	l-glycine	MSI level 2	3.569–3.556
HMDB0000177	l-histidine*	MSI level 2	3.157–3.104
			3.184–3.163
			7.081–7.045
			7.816–7.759
HMDB0000172	L-isoleucine	MSI level 1	1.018–0.100
HMDB0000687	L-leucine	MSI level 1	0.975–0.955
HMDB0000182	L-lysine	MSI level 1	3.037–3.025
HMDB0000159	L-phenylalanine	MSI level 1	7.342–7.298
HMDB0000162	L-proline*	MSI level 2	2.029–1.986
			2.337–2.278
			2.406–2.375
			3.367–3.360
HMDB0000167	L-threonine	MSI level 1	3.620–3.592
HMDB0000158	L-tyrosine	MSI level 1	6.916–6.877
HMDB0000883	L-valine	MSI level 1	1.052–1.029
N/A	Lipid	MSI level 3	4.009–3.935
N/A	Mobile lipids	MSI level 3	1.986–1.930
N/A	Mobile lipids HLDL	MSI level 3	0.800–0.793
N/A	Mobile lipids LDL	MSI level 3	0.857–0.800
N/A	Mobile lipids VLDL	MSI level 3	0.879–0.857
HMDB0000211	Myo-inositol	MSI level 1	3.592–3.569

For metabolites giving rise to multiple signals, a representative peak was selected by correlation scoring and taken forward to statistical analysis. All peaks were included for metabolites with correlation scores below 56.6% (*).

### Metabolite abundances across early pregnancy outcomes

**Figure 3:**
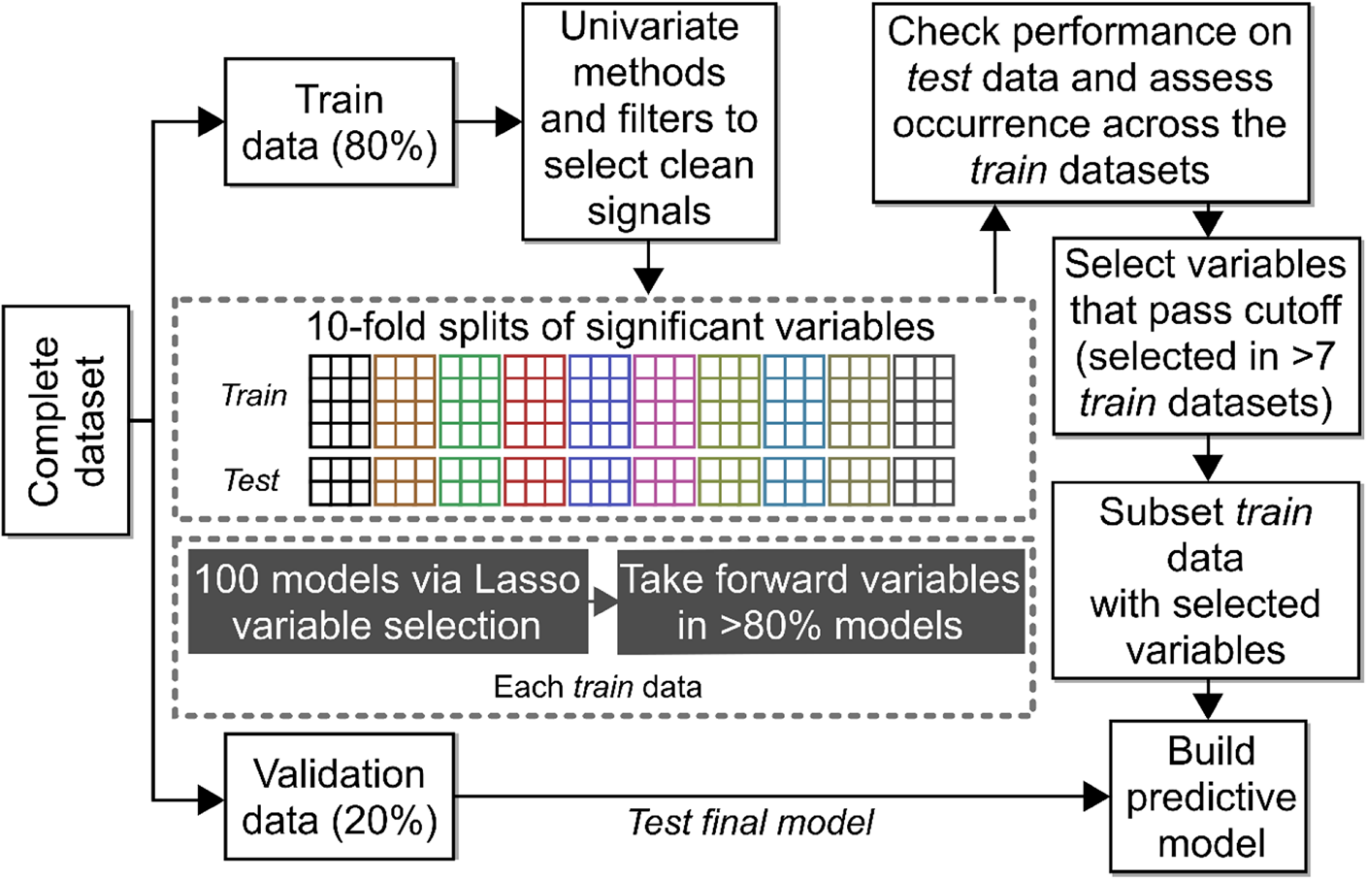
Data analysis workflow for variable selection and prediction model building.

The cohort was randomly split into discovery (80%, *n* = 253) and validation (20%, *n* = 63) sample sets (Fig. 3). Univariate analysis of the discovery set revealed 21 metabolite signals as significantly different in the LNSP group when compared with miscarriage, PUL or tEP groups (Supplementary Table 3). When miscarriage, PUL and tEP outcomes were grouped together (CAO) and compared with LNSP (Supplementary Table 4), there were 15 metabolite signals with significantly different abundances (Supplementary Table 5). Four group and two group comparisons showed 11 and five unique metabolite signals, respectively, with an overlap of 12 signals. Unsupervised PCA using plasma hormones and significant metabolite signals from univariate analysis did not demonstrate group separation (Supplementary Fig. 2).

### Selection of stable metabolite signals for biomarker identification

**Figure 4:**
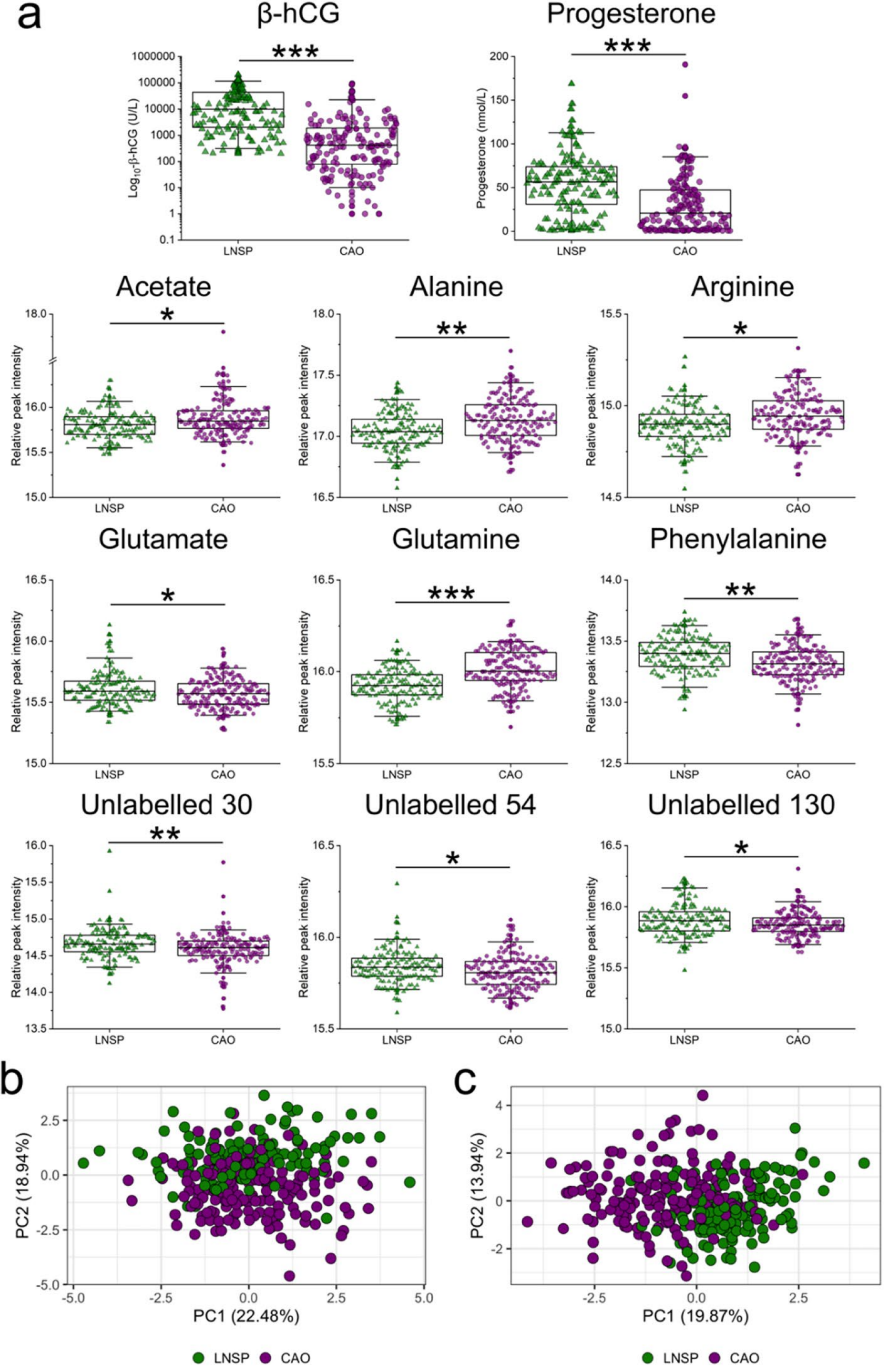
Differential abundances of biochemical markers and serum metabolites in live normally sited pregnancy (LNSP) and combined adverse outcomes (COA). (**a**) Boxplots of β-hCG, progesterone, acetate, alanine, arginine, glutamate, glutamine, phenylalanine, and unlabelled 30, 54 and 130 in LNSP and COA groups. (**b**) Principal component analysis of pregnancy outcomes using selected metabolites alone or (**c**) selected metabolites, β-hCG, progesterone, participant age, gestational age, and BMI.

The pipeline for variable selection described in the methods and Fig. 3 identified nine metabolite signals as key to discriminate LNSP from CAO using a composite marker approach: acetate, alanine, arginine, glutamate, glutamine, phenylalanine, and unlabelled signals 30, 54 and 130 (Fig. 4A). The median abundances of glutamate, phenylalanine, and unlabelled signals 30, 54 and 130 were higher in LNSP compared with CAO. Acetate, alanine, arginine, and glutamine levels were higher in the CAO group. When visualised by PCA, a discrete structure and subtle separation of groups by stable metabolite signals was apparent (Fig. 4B). Following the assessment of variance undertaken to choose the univariate analyses covariates, we also tested a model that would incorporate additional clinicodemographic variables including β-hCG, progesterone, age, BMI, and gestational age. When comparing LNSP to CAO, more clear group structure was observed in the PCA score plot (Fig. 4C). Further investigations tested signals able to discriminate between all pregnancy outcomes, but model performances were just slightly better than random (Supplementary Fig. 3A and 3B). Addition of covariates to hormone concentrations and selected metabolite signals did not significantly improved discrimination of LNSP, miscarriage, PUL and tEP groups by PCA (Supplementary Fig. 3C).

### Performance of machine learning models to predict pregnancy outcome

Random forest models were constructed for the prediction of pregnancy outcome using stable metabolite signals only or in combination with plasma hormones and clinicodemographic variables. Models were generated using the discovery cohort and performance was assessed using the independent validation cohort, which was not used for stable metabolite identification, thus preventing data leakage. Model performance was poor when all four pregnancy outcomes were included, regardless of variable inclusion (accuracy 0.48–0.58, Supplementary Table 6). When differentiating LNSP from CAO, a model with stable metabolite signals only demonstrated a modest predictive accuracy (0.68), which was comparable to a model of β-hCG and progesterone (0.71) (Table 3). The predictive accuracy of β-hCG and progesterone was marginally improved with the addition of clinicodemographic variables (accuracy = 0.73). Combining stable metabolites with hormones produced a superior model (accuracy = 0.79), while the addition of clinicodemographic variables did not improve the model further (Table 3). Clinicodemographic variables alone were unable to differentiate LNSP from CAO (accuracy = 0.44).

**Table 3:** Performance of random forest models to discriminate live normally sited pregnancy from combined adverse outcomes in the independent validation cohort.

Model	Accuracy (95% CI)	Sensitivity	Specificity	PPV	F1 score
Metabolites	0.68 (0.55–0.79)	0.68	0.70	0.72	0.70
β-hCG + P4	0.71 (0.59–0.82)	0.71	0.72	0.75	0.73
Metabolites + β-hCG + P4	0.79 (0.67–0.89)	0.76	0.83	0.84	0.80
β-hCG + P4 + age + GA + BMI	0.73 (0.60–0.83)	0.71	0.76	0.77	0.74
Metabolites + β-hCG + P4 + age + GA + BMI	0.79 (0.67–0.89)	0.74	0.86	0.86	0.80
Age + GA + BMI	0.44 (0.32–0.58)	0.59	0.28	0.49	0.54

*BMI* body mass index, *CI* confidence interval, *GA* gestational age, *β-hCG* human chorionic gonadotrophin β, *PPV* positive predictive value and *P4* progesterone.

## Discussion

We report the development of predictive models that can discriminate LNSP from multiple adverse outcomes (CAO group) at first presentation using combinations of hormone levels, metabolites and clinicodemographic features. These findings are important, since they allow for early identification of a LNSP in symptomatic women upon initial presentation to an EPAU and grants their accurate stratification for further investigations. Our study design was pragmatic to demonstrate the utility of our method in real world situations. Importantly, this study has generated a composite biomarker of early LNSP when compared with adverse pregnancy outcomes (miscarriage, PUL and tEP) using a novel machine learning pipeline. This approach has overcome the deficiencies in previous studies regarding reproducibility, while integrating untargeted ^1^H NMR small molecule profiling of serum with biochemical markers (β-hCG and progesterone) and patient demographics.

Previously examined cohorts are unlikely to be translatable to emergency clinical situations, yet a diagnostic biomarker in these scenarios would require withstanding demographic and behavioural heterogeneity (e.g., diet, exercise habits) amongst patients. Our study collected samples from symptomatic patients prior to medical assessment, including TVS, thus our data is applicable for all settings, including where access to TVS is not immediately available. In this regard, our patient population and the analytic approach are realistic and opportunistic, thus, make this data to be generalisable. Our methodology is unique in that it incorporates testing within the cohort to ensure reproducibility, whilst avoiding overfitting in the machine learning pipeline. The result is a selection of consensus variables with robust biomarker potential that should be further validated in a larger study.

Previous studies have shown that a single measurement of β-hCG cannot discriminate a LNSP from EP^27,28^. The ‘M4’ logistic regression model, which uses both the initial β-hCG concentration and β-hCG ratio to categorise PULs into low or high-risk categories^29^, performed well in the prediction of viable pregnancies as the final outcome from PULs^7^. However, its utility in a cohort of symptomatic women including miscarriage has not yet been tested. Inclusion of initial serum progesterone level in the M4 model (M6_p_ model) has been shown to improve the discrimination of EPs from failed PULs and LNSPs in a PUL cohort^13^. In agreement, meta-analysis has concluded that a low progesterone concentration (11–21 nmol/L) can exclude a LNSP with high accuracy but cannot separate EP and miscarriage cases^30^. Furthermore, previous studies have included participants beyond 10 weeks’ gestation, at which point progesterone levels are rising in viable pregnancies and likely exhibit a larger difference compared with non-viable gestations. In this study, a considerable number of participants in the LNSP group exhibited a low progesterone level, which may be due to the transient, physiological decline in progesterone between gestational weeks six to eight, corresponding to the luteal-placental shift^31^. Addition of β-hCG and progesterone to selected metabolites improved the accuracy of the prediction model. Addition of the demographic features that are known to be associated with adverse pregnancy outcomes did not improve model performance for identifying LNSPs.

We have identified perturbations in the serum metabolome pertinent to pregnancy outcome using ^1^H NMR profiling. Maternal circulatory concentrations of most essential and non-essential amino acids decrease during normal gestation, hypothetically due to placental transfer to the foetus and pregnancy adaptation for protein conservation^32^. Alanine, arginine, and glutamine levels were significantly lower in the LNSP group compared with CAO, which may reflect perturbed foetal utilisation of amino acids and thus the non-viable status of miscarriage and tEP. Furthermore, increased levels of glutamine metabolites in the endometrium have been associated with recurrent miscarriage^33^. Some amino acids, including valine, leucine, phenylalanine and glutamate have been shown to increase in early pregnancy relative to non-pregnant women^34^. Glutamate and phenylalanine levels were significantly higher in LNSP compared with CAO, thus reflecting the abnormal progression of failed LNSPs and ectopically located pregnancies. Therefore, maternal amino acid levels may represent promising biomarkers to differentiate a normal pregnancy from adverse outcomes. Importantly, serum concentrations of these metabolites can be easily measured in a clinical setting, allowing for the development of predictive algorithms that could combine biochemical markers and demographic variables at the bedside.

A study by Turkoglu et al.^35^ identified eight plasma metabolites as significantly perturbed in tEP compared with LNSP controls including acetate, lactate, and glucose. Acetate levels were increased in tEP, which agrees with our study findings, however, the remaining seven metabolites were not significantly different in our cohort. Three unlabelled metabolite signals were found to be stable markers differentiating LNSP from CAO. Unlabelled signals were those that could not be annotated from a metabolite library. It may be possible to identify these metabolites using multidimensional NMR spectroscopy or MS methods^36,37^.

Blood samples included in this study were collected from a single sex cohort with a relatively small age range, thus were expected to demonstrate any striking differences in the serum metabolome relevant to pregnancy outcome. However, these samples were subject to several metabolomic confounders including age, fasting status, medication intake, symptomology, obesity, and smoking status. BMI and smoking have been shown to induce confounding effects on the serum metabolome^38^. Age and BMI were found to contribute substantially to the observed variance in metabolite abundance between pregnancy outcomes. Several risk factors have been identified for EP, such as smoking, tubal surgery, previous EP, and sexually transmitted infections^39,40^. Risk factors for miscarriage include advanced maternal age, previous miscarriage and low or high BMI^41–43^. We did not observe differences in participant age, BMI, or smoking status between groups. Accordingly, the addition of maternal age and BMI as covariates had minimal impact on predictive accuracy in machine learning models. We excluded twin pregnancies from our LNSP group, yet any one of the CAOs may have been an undiagnosed twin pregnancy. The influence of undiagnosed twin pregnancies on the metabolomic profile would be extremely challenging to unpick, however, we would assume the impact to be very small due to the low twinning rate in our population.

In summary, this study identifies a metabolite profile associated with LNSP and incorporates this in to routinely collected hormone levels and clinicodemographic data providing predictive models that can discriminate LNSP from multiple adverse early pregnancy outcomes with reasonable accuracy. These findings may be useful in the development of a future diagnostic test to confirm a LNSP in symptomatic women and thus, exclude pregnancy loss and potentially life-threatening early pregnancy outcomes. Larger studies with independent cohorts are required to validate the accuracy, translatability and clinical utility of the predictive models described herein.

## Methods

### Study group

A total of 370 participants were recruited to the current study. These women presented as emergency clinical attendance to the Early Pregnancy Assessment Unit (EPAU) at Liverpool Women’s Hospital with symptoms of EP (abdominal pain and/or vaginal bleeding). Therefore, at this first emergency presentation, all eligible patients were symptomatic and pregnant, were ≤ 10 weeks of gestation by their last menstruation dates, without a confirmed location of pregnancy and without known final pregnancy outcome. All eligible participants self-reported to the EPAU and were triaged upon arrival. Pregnancy was confirmed with a urinary pregnancy test. Women were provided with an information leaflet, and informed written consent was obtained. Inclusion criteria comprised pregnant women aged ≥ 18 years, presenting with abdominal pain and/or bleeding at ≤ 10 weeks of gestation (calculated from last menstrual period). Women who did not meet the inclusion criteria were excluded. Demographic data were collected at the time of consent, including age and body-mass index (BMI), as well as detailed information concerning smoking status, alcohol intake, and dietary and exercise preferences. A full medical, surgical, medication and gynaecological history was also collected.

### Clinical outcomes

The final pregnancy outcomes were allocated at 12 weeks of gestation and the data were retrieved from hospital information software systems including PENS™ (Royal Liverpool University Hospitals NHS Trust, Liverpool, UK), MEDITECH™ (Westwood, MA, USA) and IDEAS™ (Mellowood Medical, Toronto, Canada) and subsequently collated. Where applicable, serial β-hCG measurements and TVS were also reviewed using Sunquest ICE™ (Sunquest Information Systems, Tucson, AZ, USA) and Picture Archiving and Communication System (PACS™) (©Carestream Health Inc, 2023, USA), respectively. Outcomes were classified in accordance with terminology guidance from the European Society of Human Reproduction and Embryology (ESHRE)^24,44^. Patients were divided into four groups: (i) LNSP; (ii) EP; (iii) miscarriage; and (iv) PUL. LNSP was defined as a pregnancy inside the uterine cavity with evident foetal heart pulsations. EP was defined as a pregnancy located outside of the uterine cavity, diagnosed either surgically at laparoscopy or on TVS. EPs were classified in line with ESHRE guidance^24^. The term miscarriage was used to describe loss of a normally sited (within the uterine cavity) pregnancy^24^. Miscarriage was diagnosed using guidance from the National Institute for Health and Care Excellence (NICE) on EP and miscarriage: diagnosis and initial management^45^. Diagnostic criteria included a mean gestational sac diameter (GSD) ≥ 25 mm with no obvious yolk sac or foetal pole on two TVS a minimum of seven days apart, or a crown-rump length (CRL) ≥ 7 mm without foetal heart pulsations on two TVS a minimum of seven days apart. Miscarriage was also diagnosed in the presence of heavy vaginal bleeding with an open cervical os, with or without products of conception. A PUL was defined as a pregnancy that had not been localised on TVS and was either treated with methotrexate or resolved spontaneously.

### Sample collection

All biosamples were processed within 1 h of collection. Blood was collected into uncoated S-Monovette^®^ Z-Gel tubes and S-Monovette® EDTA KE tubes (Sarstedt, Leicester, UK) for serum and plasma isolation, respectively. Blood in Z-Gel tubes was allowed to clot for ≥ 20 min before centrifuging at 1600×*g* for 10 min at 4 °C. EDTA KE tubes were processed immediately upon receipt; samples were centrifuged at 1600×*g* for 10 min at 4 °C. 1 mL aliquots of serum and plasma were stored in sterile cryovials at − 80 °C.

### Sample preparation

Serum aliquots were thawed and 300 μL of serum was diluted to a final volume containing 50% [v/v] serum, 40% [v/v] dd ^1^H_2_O (18.2 MΩ), 10% (v/v) 1 M PO_4_^3−^ pH 7.4 buffer (Na_2_HPO_4_, VWR International Ltd., Radnor, Pennsylvania, USA and NaH_2_PO_4_, Sigma-Aldrich, Gillingham, UK) in deuterium oxide (^2^H_2_O, Sigma-Aldrich) and 1.2 mM sodium azide (NaN_3_, Sigma-Aldrich). Samples were vortexed for 1 min, centrifuged at 21,500×*g* at 4 °C for 5 min and 600 μL transferred into 5 mm outer diameter NMR tubes.

### Spectral acquisition

Non-targeted 1D ^1^H NMR spectra were acquired at 37 °C using a 700 MHz Bruker Advance III spectrometer equipped with a TCI cryoprobe and chilled Sample-Jet autosampler (Bruker). 1D ^1^H NMR standard experiment with the cpmgpr1d filters for selective observation of low molecular weight components with optimal water suppression was acquired. Pulse sequence was vendor supplied using Carr-Purcell-Meiboom-Gill (CPMG) sequence. Serum spectra were acquired with 32 transients at 17 ppm spectral width, 72 K complex points, 3 ms echo time, 3.1 s acquisition time and a 4 s interscan delay. Full ^1^H spectrum parameter sets are available with the data deposited at MetaboLights public repository (MTBLS6219)^46^.

### Spectral processing

Automated Fourier transformation and phasing were performed in Topspin version 3.2. All spectra were individually analysed to ensure conformity with the recommended minimum reporting standards set out by the Metabolites Standard Initiative (MSI)^25,26^. Serum spectra were aligned to glucose anomeric peak at 5.244 ppm. Overall peak shapes were appraised, and average full width half maximum (FWHM) was calculated for the alignment peak; spectra with a FWHM value > 2 standard deviations from the mean were either repeated or excluded (Supplementary Table 1). No baseline corrections were applied.

### Metabolite annotation

Serum spectra were annotated using Chenomx NMR Suite 8.2 (332-mammalian metabolite library, Chenomx Inc., Edmonton, AB, Canada). Where possible, the identities of the annotated metabolites were confirmed by comparison to an in-house metabolite library in accordance with the MSI best practice. Metabolite identities were allocated to levels 1–3 of assignment confidence; level 1 required ^1^H information complemented with a secondary orthologous method (^1^H–^13^C heteronuclear single quantum coherence (HSQC), ^1^H–^1^H J-resolved, ^1^H–^1^H– correlation spectroscopy (COSY) and/or total correlation spectroscopy (TOCSY)); level 2 required matching 1D–^1^H NMR spectrum of an in-house metabolite library or external libraries available in Chenomx to the experimental spectra; level 3 comprised putatively characterised compound classes. Spectra were integrated into 162 bins with 89 annotated, corresponding to 32 unique metabolites. Annotated spectra were integrated to data matrices of peak intensities for statistical analysis. Negative intensity values, which are common in binned data^47^, were replaced with 1/5 of the minimum positive values of their corresponding variables. In order to select the most representative bin for metabolites giving rise to multiple signals, an in-house correlation reliability score (CRS) metric was utilised^48^. Correlations scores were calculated for each unique metabolite giving rise to multiple peaks, and the median score minus the standard deviation across all metabolites was set as the passing score (56.6%). When no peaks passed the CRS threshold for a given metabolite, all were included. However, such peaks are likely in overlapped regions of the spectra, therefore, metabolite annotation is of lower confidence. Spectral data is available with annotation via the MetaboLights repository (MTBLS6219)^46^.

### Progesterone and β-hCG testing

Progesterone and β-hCG concentrations were measured at the Royal Liverpool University Hospital accredited biochemistry laboratory. Plasma progesterone and β-hCG were analysed with the Elecsys Progesterone III assay and Elecsys HCG + β assay (Roche), respectively. Assays were performed on a cobas e 801 analyser (Roche) according to the manufacturer’s instructions. Assay coefficients of variability were as follows: progesterone 3 pmol/L = 10.9%, 35 pmol/L = 7.9%, 67 pmol/L = 8.1%. β-hCG: 2.9 mIU/L = 4.9%, 25 mIU/L = 5.9%, 497 mIU/L = 6.8%.

### Statistical analysis and model building

Participant age, ethnicity, BMI, smoking status, parity, gestational age, and plasma concentrations of β-hCG and progesterone were compared. Normality testing concluded that these variables did not follow a Gaussian distribution across all groups, therefore, non-parametric methods were employed. The Kruskal–Wallis test with Dunn’s multiple comparison post hoc test was used to investigate continuous variables. For categorical variables, a Chi-squared test was used. All analyses were conducted in GraphPad Prism (version 5.0). Spearman’s Rank correlation coefficients were performed in Origin Pro (version 2021b 9.85).

Metabolomics data was exploited using a univariate and multivariate statistical approach, with analyses conducted in R (version 4.1.2^49^, using in-house scripts. Data were normalised via probabilistic quotient normalisation (PQN)^50^ and log2 transformed. Technical variation/batch was assessed and removed using ComBat from the package sva^51^. This analysis can be consulted via the GitHub repository (https://github.com/EvaCaamano/ExPeDiTe_publication/tree/v1.0.0)^52^. Briefly, metabolite data was split in 80/20% train/validation sets. The train set was used to undergo variable selection firstly by a univariate statistical approach. The package limma^53^ was used to generate linear mixed models to find metabolite signals different between the groups of study considering relevant metadata (BMI, age and gestation—these covariates were chosen as they explained a significant proportion of variance of the data—see Supplementary Fig. 1) with significance adjusted for false discovery rate (FDR) using Benjamini and Hochberg^54^. Significant signals at 5% FDR were taken forward for multivariate selection. The train dataset was subjected to a tenfold cross-validation in a 90/10% split, where each 90% split underwent 100 rounds of Least Absolute Shrinkage and Selection Operator (LASSO, glmnet package^55^) selection. Signals that were selected in at least 80% of the rounds and more than 8 folds were taken forward for modelling. Random forest models^56^ were constructed with selected metabolites and metadata variables and performances were assessed in both train and validation datasets. Further tests were done by building generalised linear models and calculating area under the receiver operator characteristic curves (AUROCs).

Data visualisation was performed with Origin Pro and R package ggplot2^57^. Significant *p* values are highlighted with asterisks (*p* < 0.05*, *p* < 0.01**, *p* < 0.001***).

### Ethical approval

This research study has been approved by Liverpool Women’s Hospital Research & Development Team and the North West Liverpool Central Research Ethics Committee (REC 17/NW/0646).

### Supplementary Information

Supplementary Information.

## Data Availability

NMR data is available via the EMBL-EBI MetaboLights repository (ID: MTBLS6219). Code and analyses for all main results are available via GitHub repository link https://github.com/EvaCaamano/ExPeDiTe_publication/tree/v1.0.0^52^.

## Abbreviations

BMI: Body mass index
CI: Confidence interval
CAO: Combined adverse outcomes
CRS: Correlation reliability score
CRL: Crown rump length
EP: Ectopic pregnancy
FDR: False discovery rate
GSD: Gestational sack diameter
β-hCG: Human chorionic gonadotrophin β
IQR: Interquartile range
LNSP: Live normally sited pregnancy
NVNSP: Non-viable normally sited pregnancy
NMR: Nuclear magnetic resonance
PUL: Pregnancy of unknown location
PPV: Positive predictive value
POC: Products of conception
PCA: Principal component analysis
P4: Progesterone
TVS: Transvaginal ultrasound
tEP: Tubal ectopic pregnancy
