# Supplementary material for: Diagnostic utility of clinicodemographic, biochemical and metabolite variables to identify viable pregnancies in a symptomatic cohort during early gestation
Source: Sci Rep. 2024 May 15;14:11172. doi: 10.1038/s41598-024-61690-3 (PMC11096363; doi:10.1038/s41598-024-61690-3)
Supplement: Supplementary file 1 — Supplementary Information. [file 41598_2024_61690_MOESM1_ESM.pdf]

Supplementary Information

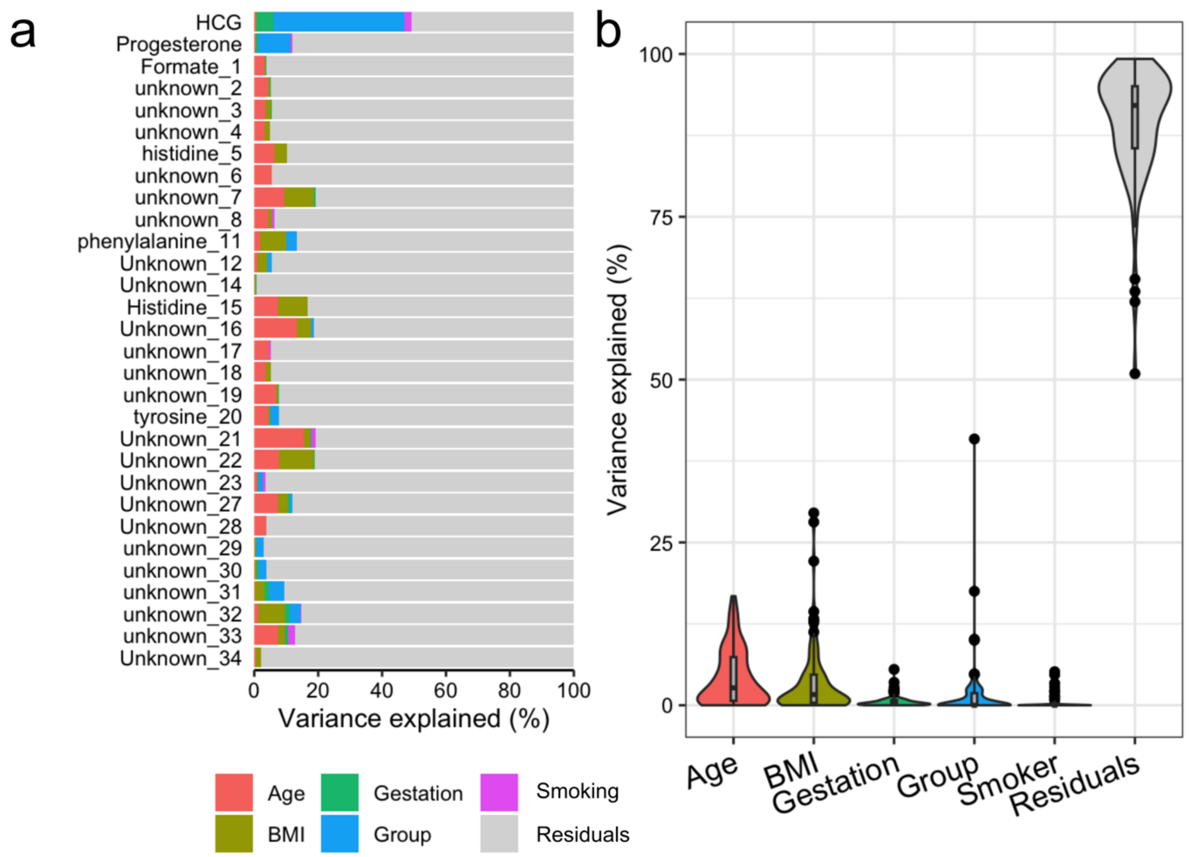

Supplementary Figure 1. Analysis of variance to determine covariates. Abbreviations: BMI (body mass index) and human chorionic gonadotrophin β (HCG).

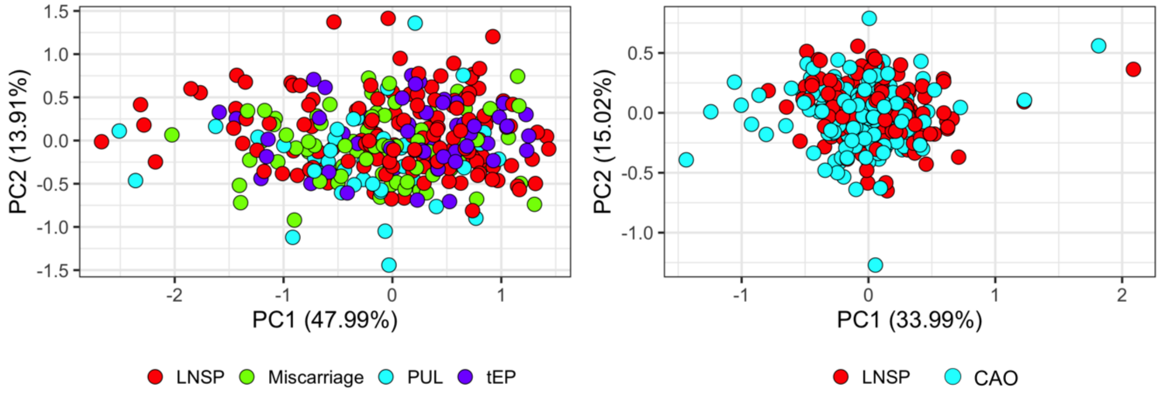

Supplementary Figure 2. Principal component analysis of pregnancy outcome using plasma hormone concentrations (β-hCG and progesterone) and differentially abundant metabolite signals identified by univariate analysis.

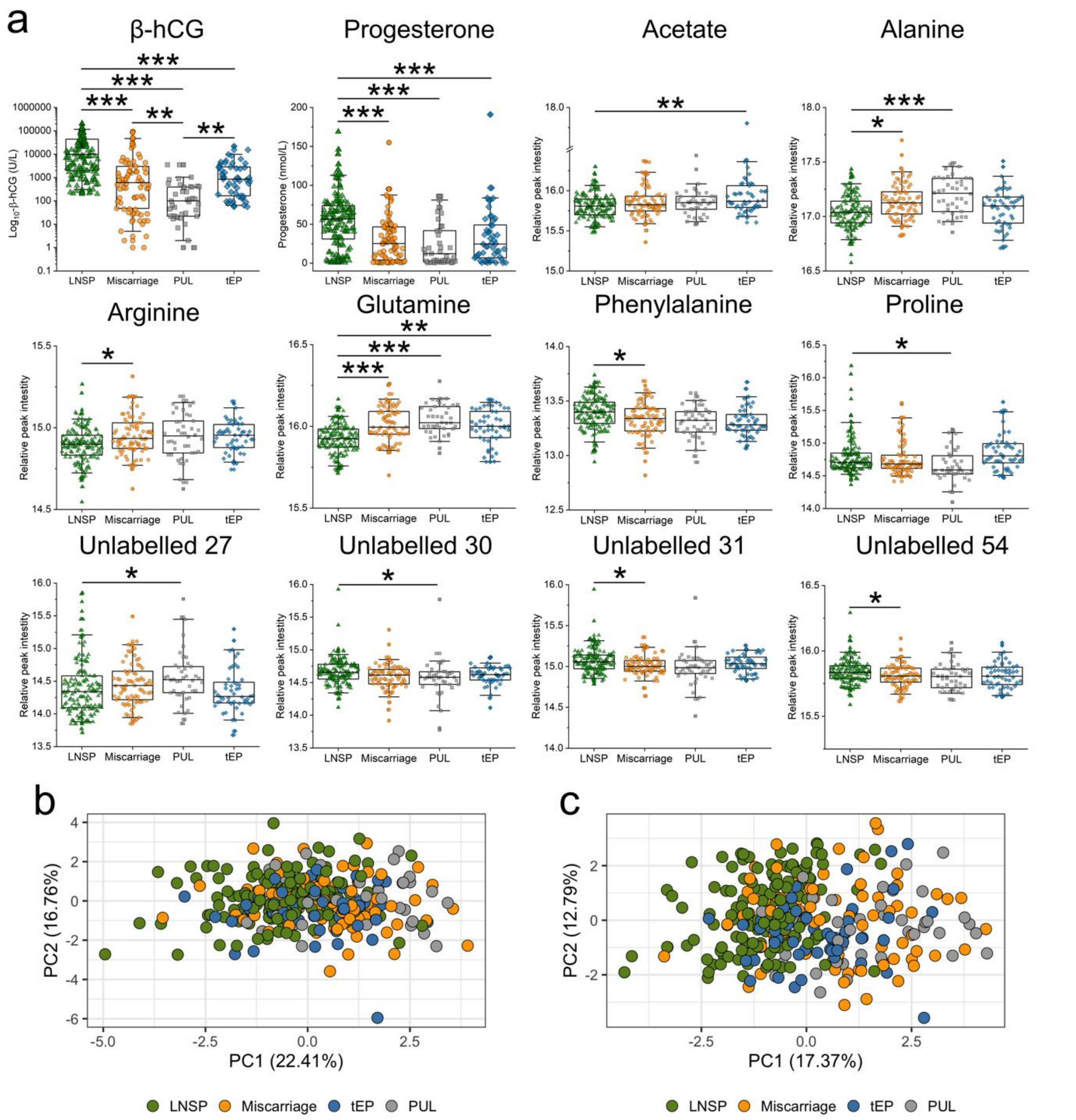

Supplementary Figure 3. Comparison of biochemical markers and serum metabolite abundances across all pregnancy outcomes. (a) Boxplots of β-hCG, progesterone, acetate, alanine, arginine, glutamine, phenylalanine, proline, and unlabelled 27, 30, 51 and 54 in live normally sited pregnancy (LNSP), miscarriage, pregnancy of unknown location (PUL) and tubal ectopic pregnancy (tEP) groups. (b) Principal component analysis of pregnancy outcomes using selected metabolites and (c) selected metabolites with β-hCG, progesterone, age, gestational age, and BMI.

Supplementary Table 1. Participants excluded from analysis. Abbreviations: ethylenediaminetetraacetic acid (EDTA), live normally sited pregnancy (LNSP), pregnancy of unknown location (PUL), quality control (QC) and tubal ectopic pregnancy (tEP).

Study ID	Outcome	Reason for exclusion
ET014	Miscarriage	Missing metadata
ET018	PUL	Failed QC
ET043	Vanishing twin syndrome	Pregnancy outcome
ET048	Vanishing twin syndrome	Pregnancy outcome
ET057	LNSP	Failed QC
ET071	NA	Outcome unknown
ET073	NA	Outcome unknown
ET083	Miscarriage	Missing metadata
ET093	PUL	Missing metadata
ET097	NA	Outcome unknown
ET106	PUL	Missing metadata
ET133	LNSP	Failed QC
ET140	LNSP	Failed QC
ET151	Miscarriage	EDTA contamination
ET154	PUL	Failed QC
ET166	Miscarriage	EDTA contamination
ET168	Miscarriage	Failed QC
ET169	tEP	Failed QC
ET170	LNSP	Failed QC
ET171	Miscarriage	Failed QC
ET173	PUL	Missing data
ET176	LNSP	EDTA contamination
ET191	Miscarriage	Missing metadata
ET196	LNSP	Missing metadata
ET208	LNSP	Missing metadata
ET210	Miscarriage	Failed QC
ET229	NA	Outcome unknown
ET230	Miscarriage	Failed QC
ET245	Miscarriage	Failed QC
ET249	Miscarriage	Failed QC
ET251	LNSP	Missing metadata

ET254	NA	Outcome unknown
ET259	Twin pregnancy	Pregnancy outcome
ET271	tEP	Missing data
ET279	Miscarriage	Missing data
ET281	Twin pregnancy	Pregnancy outcome
ET299	Miscarriage	Failed QC
ET315	Not pregnant	Pregnancy outcome
ET316	Miscarriage	Failed QC
ET325	Miscarriage	Failed QC
ET350	C-section ectopic	Pregnancy outcome
ET361	LNSP	Failed QC
ET369	NA	Outcome unknown
ET373	tEP	High gluconic acid
ET383	NA	Outcome unknown
ET384	Not pregnant	Pregnancy outcome
ET392	NA	Outcome unknown
ET394	LNSP	Failed QC
ET398	NA	Outcome unknown
ET406	tEP	Missing metadata
ET426	Ovarian ectopic	Pregnancy outcome

Supplementary Table 2. Spearman's rank correlation coefficients for plasma β-hCG and progesterone concentrations, and gestational age.

	**β-hCG – gestational age**		**Progesterone – gestational age**		**β-hCG – progesterone**	
	ρ	*p*-value	ρ	*p*-value	ρ	*p*-value
LNSP	0.59	<0.0001	-0.12	0.13	-0.17	0.52
Miscarriage	0.33	0.05	0.07	0.90	0.15	0.35
PUL	0.21	0.05	0.12	0.81	0.28	0.08
tEP	0.01	0.89	-0.17	0.40	-0.01	0.58

Supplementary Table 3. Serum metabolite signals with significantly different abundances between pregnancy outcomes. Signals that are unique to the four-group comparison are highlighted (*). Abbreviations: false discovery rate (FDR), live normally sited pregnancy (LNSP), pregnancy of unknown location (PUL), and tubal ectopic pregnancy (tEP).

Comparison	Metabolite	FDR-adjusted *p* value
LNSP-Miscarriage	2-hydroxybutyrate*	0.04
	Alanine	0.04
	Arginine	0.04
	Glutamine	>0.001
	Phenylalanine	0.04
	Unlabelled 31	0.04
	Unlabelled 54	0.04
LNSP-PUL	2-hydroxybutyrate*	0.04
	2-hydroxyisovalerate*	0.02
	Alanine	>0.001
	Creatinine*	0.005
	Glutamine	>0.001
	LDL*	0.03
	Proline*	0.01
	Tyrosine*	0.04
	Unlabelled 27*	0.01
	Unlabelled 29	0.03
	Unlabelled 30	0.03
	Unlabelled 31	0.003
	Unlabelled 32*	0.02
	Unlabelled 52*	0.01
	Unlabelled 99*	0.04
	Unlabelled 120	0.03
LNSP-tEP	Acetate	0.003
	Glutamine	0.003

Supplementary Table 4. Comparison of participant demographics in the live normally sited pregnancy (LNSP) and combined adverse outcomes (CAO) groups. Abbreviations: body mass index (BMI), human chorionic gonadotrophin β (β-hCG), interquartile range (IQR).

	LNSP	CAO	*p*-value
Number	146	170	
Age in years, median (IQR)	30 (8)	30 (9)	0.19[a]
Caucasian ethnicity, *n* (%)	127 (87.0)	138 (81.2)	0.20[b]
BMI (kg/m^2), median (IQR)	25.8 (7.9)	24.7 (8.1)	0.25[a]
Smoker, *n* (%)	36 (24.7)	39 (23.0)	0.74[b]
Nullipara, *n* (%)	54 (37.0)	62 (36.4)	0.81[b]
Gestational age in weeks, median (IQR)	6 (2)	6 (2)	0.02[a]
β-hCG U/L, median (IQR)	9893 (42310)	416 (1902)	<0.001[a]
Progesterone nmol/L, median (IQR)	57 (44)	21 (44)	<0.001[a]

[a]Mann-Whitney U test [b]Chi-squared test

Supplementary Table 5. Serum metabolite signals with significantly different abundances between live normally sited pregnancies and combined adverse outcomes. Signals that are unique to the two-group comparison are highlighted (*). Abbreviations: false discovery rate (FDR).

**Metabolite**	**FDR-adjusted *p* value**
Acetate	0.04
Alanine	0.003
Arginine	0.01
Glutamate*	0.02
Glutamine	>0.001
Phenylalanine	0.007
Unlabelled 29	0.01
Unlabelled 30	0.008
Unlabelled 31	0.007
Unlabelled 54	0.02
Unlabelled 57*	0.04
Unlabelled 88*	0.008
Unlabelled 102*	0.01
Unlabelled 120	0.03
Unlabelled 130*	0.01

Supplementary Table 6. Performance of random forest models to predict pregnancy outcome in the independent validation cohort. Abbreviations: body mass index (BMI), confidence interval (CI), gestational age (GA), human chorionic gonadotrophin β (β-hCG), positive predictive value (PPV) and progesterone (P4).

Model		*LNSP*	*Miscarriage*	*PUL*	*tEP*
Metabolites	Accuracy (95% CI)		0.48 (0.36-0.61)		
	Bal. accuracy	0.73	0.57	0.46	0.48
	Sensitivity	0.82	0.33	0.00	0.10
	Specificity	0.64	0.81	0.93	0.87
	PPV	0.67	0.36	0.00	0.13
	F1 score	0.74	0.34	0.00	0.11
Metabolites + β-hCG + P4	Accuracy (95% CI)		0.56 (0.43-0.69)		
	Bal. accuracy	0.77	0.61	0.53	0.53
	Sensitivity	0.93	0.40	0.13	0.10
	Specificity	0.61	0.83	0.93	0.96
	PPV	0.68	0.43	0.20	0.33
	F1 score	0.79	0.41	0.16	0.15
Metabolites + β-hCG + P4 + age + GA + BMI	Accuracy (95% CI)		0.58 (0.45-0.70)		
	Bal. accuracy	0.82	0.63	0.59	0.46
	Sensitivity	0.97	0.40	0.25	0.00
	Specificity	0.67	0.85	0.93	0.92
	PPV	0.72	0.46	0.33	0.00
	F1 score	0.83	0.43	0.28	0.00
β-hCG + P4 + age + GA + BMI	Accuracy (95% CI)		0.55 (0.42-0.68)		
	Bal. accuracy	0.80	0.55	0.56	0.49
	Sensitivity	0.97	0.27	0.25	0.00
	Specificity	0.64	0.83	0.87	0.98
	PPV	0.70	0.33	0.22	0.00
	F1 score	0.81	0.30	0.23	0.00
